# A theory of degrees in education

**DOI:** 10.1016/j.heliyon.2024.e36008

**Published:** 2024-08-08

**Authors:** Stefan Winter, Melissa Kistner, Deborah Maffia

**Affiliations:** Ruhr-University Bochum, Germany

**Keywords:** Degree, Education, Technological platform, Economic theory, Optimization

## Abstract

Degrees are presumably the most important institution in education markets. However, despite their high relevance, degrees and their design features remain poorly understood within the economics of education. This study addresses this gap. We provide an accurate formal definition of what constitutes a degree and formulate the first theory of degree design. This theory is based on a technological platform view that interprets degrees as modules within a hierarchical structure resembling that of software architectures. This approach provides new opportunities to address questions of degree optimization. Furthermore, a novel strategy to optimize the design of school degrees is proposed. This strategy is expected to improve the learning environment of weaker students across all school types, leaving higher-performing students rather unaffected. The implications and limitations of our approach are also discussed.

## 1 Introduction

At present, the most important building blocks of formal education, such as school education, are organized in the form of educational degrees. However, despite their high relevance, degrees remain poorly understood. In fact, literature on the economics of education does not provide a clear definition of what constitutes a degree. Neither textbooks on the economics of education nor labor economics textbooks discussing the role of education provide any definitions at all (e.g., Refs. [[1], [2], [3], [4], [5]]). Considering that degrees are the dominant form of educational organization worldwide, it is more than astonishing that not even definitions of degrees exist. Thus, the first objective of this study is to develop an initial and precise definition of what constitutes a degree.

As degrees have not yet been defined, it is not surprising that the rationales for their existence and design features are also not well understood. So far, theories within the economics of education, particularly human capital theory and the signaling approach, can only explain why people want to invest in education. However, they cannot explain why people invest by obtaining degrees. According to the human capital theory [6,7], education enhances productivity, which then translates into higher wages in competitive labor markets. According to the signaling approach [8], education serves as a signal of superior but hidden productivity characteristics in markets characterized by asymmetric information. Prospective employees get higher wage offers after obtaining more education. It is expected that those with more education are more productive. In equilibrium, this expectation holds true.

However, the human capital theory and the signaling approach are not mutually exclusive. Spence [9] demonstrated that signaling may still play a role even if education at the same time improves productivity. The advantages of the aforementioned approaches have been intensely scrutinized and both found support [10]. Exactly disentangling the human capital and signaling effects is considered to be difficult or even impossible [11]. However, with respect to the aim of this study, both theoretical approaches share an important commonality. Both treat education as a one-dimensional quantity variable that can be acquired in marginal units. Neither of them provides a rationale for the existence of degrees and can explain the design features of educational degrees.

Other economic approaches also do not address the existence or fundamental design issues of degrees. Within the empirical literature on the economics of education, only two branches explicitly address the role of degrees. The returns to education stream finds that better education is associated with higher earnings (e.g., Refs. [[12], [13], [14], [15]]). Within that stream, some studies explicitly control for degree levels and/or subjects (e.g., Refs. [[16], [17], [18], [19], [20]]). The second stream is concerned with the so-called sheepskin effect, postulating that returns to a year of education in which a degree is earned are higher than returns to years of education in which no degree is acquired [21]. Although some studies have found no or, in some cases, only minor effects (e.g., Refs. [21,22]), the majority of studies conclude that there are indeed sheepskin effects (e.g., Refs. [[23], [24], [25], [26]]). However, both streams treat degrees just as given empirical phenomena. Explaining why degrees exist and why they are designed the way they are is again beyond the aim of both approaches. Degrees are treated as black boxes.

We thus conclude that the concept of a degree is not well understood theoretically or empirically. Therefore, the second objective of this study is to develop the first economic theory that explains the existence of degrees and their design features. This theory helps advance research on education in that it allows to ask completely new questions regarding degree design.

Because degrees and their design features are not well understood, it is again not surprising that educational reforms treat such design features as given and try to optimize within the existent design. Typical reforms address variations of class size (e.g., Refs. [[27], [28], [29], [30]]), improve teaching quality (e.g., Refs. [[31], [32], [33], [34], [35]]), or add tutoring (e.g., Refs. [[36], [37], [38], [39], [40]]). By optimizing within the given design, the potential efficiency gains from changing the basic design features are foregone. Applying the theory developed here, the third objective of this study is to derive an alternative, fundamentally different degree design that allows capturing of such design-dependent efficiency gains. We expect the efficiency gains to be disproportionally high for weaker students.

To summarize, our study aimed to a) provide the first precise scientific definition of what constitutes a degree, b) develop the first economic theory that explains the inner complexities and design features of degrees, and c) use that theory to develop an alternative, optimized degree design.

Our study is a purely theoretical contribution. The main limitation of our conclusions is the lack of direct empirical support. However, indirect evidence that corroborates our suggestions is available. Fully testing our suggestions is straightforward but would require a multiyear field experiment.

The remainder of this paper is organized as follows: In Section 2, we develop the first formal definition of what constitutes a degree. This definition is based on stylized facts of the most important design features of typical degrees. In Section 3, we describe the basic characteristics of product platforms and apply the technological platform view to develop the first theory of educational degrees. In Section 4, we demonstrate how the technological platform view can be used to develop novel strategies to optimize the design of school degrees. In Section 5, we discuss further implications. Then, in Section 6, we outline the limitations of our suggestion. Finally, we provide the conclusion in Section 7.

## 2 Definition

While the term degree is regularly utilized in educational contexts, precise scientific definitions are lacking. This is somewhat surprising considering that degrees are presumably the most important product traded in markets for education. Within the scientific literature, Mause [41] is the only one we were able to identify who has provided at least a vague definition. Accordingly, a degree is a certificate, edited by a school or university, confirming that the certificate holder has acquired knowledge within some field of interest. Certification only takes place after the certificate holder has met at least the minimum threshold requirements of the corresponding examinations [41].

A nonscientific source provided another definition as follows: a degree is any of a wide range of status levels conferred by institutions of higher education, such as universities, usually as a result of successful completion of a program of study [42]. A similar nonscientific definition states that *An academic degree is a position and title within a college or university that is usually* awarded *in recognition of the recipient having either satisfactorily completed a prescribed course of study or having conducted a scholarly endeavor deemed worthy of his or her admission to the degree. The most common degrees* awarded *today are Bachelor's, Master's, and doctoral degrees* [43].

The commonality of these definitions is that degrees are awarded for success in examinations that cover more than just one topic/subject, but rather cover a “bundle” or “program of study”. These bundles may be defined by requirements of a certain discipline or job the program is aimed at [44].

The following working definition is based on stylized facts of what is typically called a “degree” in practice. Without further inquiry, we assume that the most important degrees are school and college/university degrees and refer to these in the following.

### 2.1 Scope

The first stylized fact about degrees is that to earn one of them, completion of a predefined set of courses is required, so there is a bundle or program. An immediate implication is that degrees cannot be obtained in marginal units. In the following, we assume that the individual course is the basic unit of those bundles or programs. Programs are thus characterized by their scope of courses. This scope of courses may consist of two distinct course sets, i.e., a mandatory and a voluntary choice course set. Students must take all courses of the mandatory course set and a subset of the choice course set.

Let $M_{j}$ be the set of mandatory courses $c_{m}$ that must be successfully completed to obtain the degree in discipline j. For example, a bachelor degree in discipline j = economics may require successful completion of courses on m = statistics, mathematics, microeconomics, and others. Let $n_{m}=n\left(c_{m}\right)$ be a binary variable with $n_{m}=1$ indicating that course $c_{m}$ has been successfully completed and $n_{m}=0$ otherwise. Let $N_{jM}$ be the number of courses in course set $M_{j}\text{.}$ Condition $C_{1}$ for obtaining a degree can then be expressed as follows:

(1) $$C_{1}:\sum_{m}n_{m}=N_{jM}$$

Now, there may be a second set of courses that allows for individual course choices. Let $V_{j}$ be the set of courses $c_{v}$ that can be freely chosen within a program in discipline j. For example, students may be offered a set of several choice courses. While the courses themselves can be freely chosen from that set, there is also an obligation to select a specified number of courses from that set. Therefore, the choices made are not entirely voluntary. Students are allowed to select or disregard any single course of that set, but they must also select some of the courses. Let $n_{v}=n\left(c_{v}\right)$ be a binary variable with $n_{v}=1$ indicating that course $c_{v}$ has been successfully completed and $n_{v}=0$ otherwise. Let $N_{jVmin}$ be a positive integer identifying the minimum number of courses that must be selected and successfully completed from the set $V_{j}$.

One further stylized fact of degrees is that there are not only minimum thresholds in the number of courses to be successfully completed but also upper limits. There is no unlimited “not yet” option for students. They cannot just decide that they are not yet willing to accept the degree and, instead, continue to take additional courses forever. There might be small corridors available, though. Let $N_{jVmax}$ be a positive integer identifying the maximum number of courses that can be selected and successfully completed from the set $V_{j}$. Therefore, condition $C_{2}$ for obtaining a degree can then be expressed as follows:

(2) $$C_{2}:N_{jVmin}\leq \sum_{v}n_{v}\leq N_{jVmax}$$

Conditions $C_{1}$ and $C_{2}$ represent the scope conditions, as these conditions specify the scope of topics/subjects covered by the degree in discipline *j*. This scope may be related to some disciplines in the narrow sense of disciplines, such as economics, law, and medicine, or the scope may cover rather unrelated subjects such as in school degrees. The scope of some degree may be completely defined by a mandatory course set, a voluntary course set, or a combination of both. School degrees typically offer little if any individual choices, whereas college and university degrees may completely comprise voluntary course sets.

### 2.2 Quality, standardization, and compatibility

If human brains would be hardware and course contents would be software and if both hardware and software would be completely standardized and compatible, then the aforementioned scope criteria would suffice to define a degree. All course contents would just be installed on all students’ brains alike, and all students with a degree in the same program would have gained the same knowledge. However, human brains and human knowledge are not standardizable to that extent. There is thus some room for interindividual differences in degree quality. Therefore, quality differentiation is the next defining characteristic of degrees.

For quality differentiation, a procedure called examination is invoked. The result of that procedure is a quality of success rating called grade. Let $e_{i}$ be the student's output in an examination of course $c_{i}$. We assume that only one examination per course is held. The student's output is rated on an alpha-numeric grading scale by an individual typically called teacher, professor, or lecturer. Let $g\left(e_{i}\right)$ be the numerical grade awarded to the student after delivering examination output $e_{i}$. In what follows, it is assumed that grading is correctly done without error and that grades are weakly increasing in student output. A course is successfully completed if the grade $g\left(e_{i}\right)$ meets or exceeds the required grade minimum $g_{\min}$. Again, let $n_{i}=n\left(c_{i}\right)$ be a binary variable with $n_{i}=1$ indicating that course $c_{i}$ has been successfully completed and $n_{i}=0$ otherwise. Considering the grading procedure, the condition of successful course completion thus reads $n_{i}=1$ if $g\left(e_{i}\right)\geq g_{\min}$, whereas failing is indicated by $n_{i}=0$.

The next stylized fact is that the number of possible examinations not meeting the threshold requirements is limited. Let $f\left(c_{i}\right)$ be the number of attempted examinations in course $c_{i}$ in which the minimum requirement $g\left(e_{i}\right)\geq g_{\min}$ is not met. There is then a critical threshold value $F\left(c_{i}\right)$, so that if $f\left(c_{i}\right)\;>\;F\left(c_{i}\right)$, the student must leave the program without earning the degree. Usually, the critical value F is independent of i, so that $F\left(c_{i}\right)=F\;\forall i\text{.}$ However, being excluded from the program after too many fails may be limited to examinations in mandatory courses. Again, let $c_{m}$ be any of the courses in the mandatory course set $M_{j}$. Condition 3 for earning a degree can thus be expressed as follows:

(3) $$C_{3}:∄\;m:f\left(c_{m}\right)\;>\;F\left(c_{m}\right)$$

Variations of this condition are also possible. For example, there may be an examination budget B, i.e., an upper limit of the total number of examinations across all courses. Thus, if $\sum_{i}f\left(c_{i}\right)\;>\;B$, the student must leave the program without earning a degree. In some cases, there may also be a budget of final fails, so that a degree is awarded even when a student has finally failed in some courses if the total number of fails does not exceed the limit of the budget.

The next stylized fact is that not only single courses are graded but also degrees themselves. Furthermore, grades are enforced on students, i.e., students cannot opt to receive a nongraded degree. Let $D_{jk}=1$ indicate that student k has earned a degree in discipline j. Let $G_{k}\left(D_{jk}\right)$ be student k’s grade of that degree. The grade of the degree $G_{k}\left(D_{jk}\right)$ is typically calculated as a weighted average of the grades $g_{ik}=g_{k}\left(c_{i}\right)$ of the individual courses taken, i.e., $G_{k}\left(D_{jk}\right)=\sum_{i}w_{i}g_{ik}$, where $w_{i}$ denotes the weight of grade $g_{ik}$ achieved in course $c_{i}$. Total grades G and individual course grades g typically use the same scales. The weights $w_{i}$ themselves are usually defined by some course quantity measures such as workload, not by quality measures such as difficulty or complexity. Difficulty and complexity are rather used to select the degree level the course is assigned to. That absence of opting out of grading represents our fourth degree condition: If a degree is awarded, then that degree is graded and that grade is made of a weighted average of course grades. Condition $C_{4}$ thus reads as follows:

(4) $$C_{4}{:if\;D}_{jk}=1:\exists G_{k}\left(D_{jk}\right)\;so\;that\;G_{k}\left(D_{jk}\right)=\sum_{i}w_{i}g_{ik}$$

### 2.3 Modular hierarchical embeddedness

Degrees exhibit a modular design. There is not one degree covering education from the age of 6 up to a degree in law at the age of 23 and another degree covering education from the age of 6 up to a degree in medicine at the age of 25. Instead, lawyers, physicians, economists, and all others share the core module called school degree, covering education from the age of 6 up to finishing high school. Only after high school do lawyers, physicians, and economists complement their high school degrees with specialized degrees in their respective disciplines.

This modular design of educational degrees is at the same time hierarchical. Thus, the next stylized fact about degrees is that they are hierarchically ordered. One can only enter college after successfully completing high school, and one can only enter a PhD program after completing a master (sometimes bachelor) program. Let $D^{L}$ be an indicator variable. $D_{jk}^{L}=1$ indicates that individual k has earned a degree of level L in discipline j. If k has not earned that degree, $D_{jk}^{L}$ equals zero. Hierarchical ordering means that before a student can enroll in a program to obtain a level L degree, a degree of level L−1 has to be earned. Let $Z_{j}^{L-1}$ be the set of feasible degrees $D_{z\left(j\right)}^{L-1}$ of level L−1 that offers individual k the opportunity to enter a program to earn the next-level degree $D_{j}^{L}$. Individual k must have obtained at least one of the degrees out of the degree set $Z_{j}^{L-1}$. Thus, the fifth general condition $C_{5}$ to earn a degree $D_{jk}^{L}$ can be written as follows:

(5) $$C_{5}:{\exists \;z\left(j\right):D}_{z\left(j\right)k}^{L-1}=1$$

Variable z(j) denotes some discipline as variable j. While at least one level L−1 degree in some discipline z(j) is required to enter a program for a level L degree in discipline j, disciplines z(j) and j may coincide or differ. A school degree $D_{z\left(j\right)}$ that is required to obtain access to colleges or universities is typically a degree that covers a broad range of subjects and is thus not a degree in some narrowly defined discipline. If a bachelor program in j= economics requires a high school degree z(j), then j and z(j) differ; j is a narrowly defined discipline, whereas z(j) is not. Meanwhile, some master programs require completion of a bachelor program in the same discipline so that j and z(j) coincide. z(j) may or may not depend on j. An example for the latter is the Abitur $D_{z}$, i.e., the German university entrance degree, which gives access to any discipline j offered at German public universities, so that z(j)=z ∀j.

The initial pseudo-degree $D_{z}^{0}$ in this hierarchical system of degrees can be simply defined as a child's ability to join an elementary school. This nonformal school entrance degree is usually just defined by age, combined with the assumption that the child has the intellectual capacities to enter primary school.

Condition $C_{5}$ is stated as a binary yes/no criterion of access solely based on the existence or nonexistence of the lower-level degree. In addition, there may also be grade restrictions. It might not suffice to have obtained the lower-level degree $D_{z\left(j\right)}^{L-1}$ to obtain access to the next-level program. Instead, it might also be required that the grade of the lower-level degree, i.e., ${G(D}_{z\left(j\right)}^{L-1})$ must at least meet some minimum threshold value $G_{\min}$. Access to some college might not only require a high school diploma but also a good High School Grade Point Average (HSGPA). That threshold grade may depend on the next-level degree to be obtained, so that ${G_{\min}=G}_{\min}\left(D_{j}^{L}\right)$. For example, the grade requirements to obtain access to a program in medicine are typically tougher than the requirements for programs in the social sciences. Thus, condition $C_{6}$ can be written as follows:

(6) $$C_{6}:{\exists \;z\left(j\right):G(D}_{z\left(j\right)k}^{L-1})\geq G_{\min}\left(D_{j}^{L}\right)$$

The embeddedness criteria $C_{5}$ and $C_{6}$ may not be exhaustive to describe access restrictions. There may be other institutions involved, such as admission tests. There could also be special weightings of single-course grades. For example, a college offering a physics program might be more interested in math grades than in HSGPA. Furthermore, outside of the degree programs, there may be special courses to better prepare students for later educational stages. Such courses can be interpreted as an adapter technology to improve compatibility between stages.

While the scope criteria $C_{1}$ and $C_{2}$ and the quality criteria $C_{3}$ and $C_{4}$ refer to conditions that must be met to earn a degree after joining some degree program, the embeddedness criteria $C_{5}$ and $C_{6}$ refer to conditions that must be met to join such a degree program. Table 1 presents a brief summary of $C_{1}-C_{6}$ with explanatory notes.

**Table 1:** Overview of degree conditions.

**Scope**		
C_1_:	$\sum_{m}n_{m}=N_{jM}$	C_1_ states that all mandatory courses must be successfully completed.
C_2_:	$N_{jVmin}\leq \sum_{v}n_{v}\leq N_{jVmax}$	C_2_ states that students must take and successfully complete a subset of courses from the voluntary course set. The number of courses may allow for some variations. However, the number of courses must surpass a minimum threshold and not exceed an upper limit.
**Quality, Standardization, and Compatibility**		
C_3_:	$∄\;m:f\left(c_{m}\right)\;>\;F\left(c_{m}\right)$	C_3_ states that students must leave a program without obtaining the degree if the number of failed examinations in mandatory courses exceeds a predetermined threshold.
C_4_:	${if\;D}_{jk}=1:\exists G_{k}\left(D_{jk}\right)\;so\;that\;G_{k}\left(D_{jk}\right)=\sum_{i}w_{i}g_{ik}$	C_4_ states that if a degree is awarded, then that degree is graded, and that grade is made of the weighted average of course grades. Students cannot decide to receive an ungraded degree.
**Modular Hierarchical Embeddedness**		
C_5_:	${\exists \;z\left(j\right):D}_{z\left(j\right)k}^{L-1}=1$	C_5_ states that students must obtain at least one of the accepted lower-level degrees to obtain access to a next-level degree program. For example, students must obtain a high school degree before they are admitted to college programs.
C_6_:	${\exists \;z\left(j\right):G(D}_{z\left(j\right)k}^{L-1})$ ≥ $G_{\min}\left(D_{j}^{L}\right)$	C_6_ further states that eventually the grade of the accepted lower-level degree must meet at least the minimum threshold value to obtain access to the next-level program. For example, access to a program in medicine may not only require a high school degree but rather a good one.

The next stylized fact is the existence of institutions that offer degrees, such as schools, colleges, or universities. These institutions can be interpreted as transaction platforms coordinating supply of education services from teachers and demand for those from students. For the purpose of this study, it suffices to say that these institutions award degrees.

Another stylized fact about degrees is that the mandatory course set $I_{jM}$ and the voluntary course set $I_{jV}$ are fixed ex ante and do not depend on the individual. If there is a mandatory course set, it is the same for all students in the program. The same is true for the grading functions used and the evaluation procedures. These may be subject to teachers’ subjectivity, though. However, from a theoretical point of view, evaluation procedures are meant to be independent of the individuals involved. Evaluation results should depend on performance only and not on any other performance-unrelated criteria. The hierarchically ordered sequence of degrees to pass through is also the same for all. Thus, degrees themselves and their hierarchical ordering exhibit a high level of standardization. Degrees are also typically offered in a standardized time frame. This indicates that institutions offering degrees guarantee that students can obtain the degree within a specified time frame. Courses, seminars, and examinations are offered in a time schedule that enables students to complete the degree within that span. Students may choose to complete the degree faster or slower, but at the time of enrollment, they are at least implicitly guaranteed the opportunity to earn the degree within the specified time frame.

Taken together, these stylized facts lead to our following working definition: A level L degree of individual k in discipline j is a certified verification that k has met $C_{1}-C_{6}$. Grading procedures, time frames, and course sets are highly standardized. The certifying institutions are transaction platforms, which are typically called schools, colleges, or universities.

In the following, we discuss the design characteristics of technological platforms. The technological platform design can be used as a framework that provides understanding of the advantages of designing degrees the way they are designed, as discussed above. The technological platform view also offers new opportunities to address questions of degree optimization.

## 3 Technological platform view

What are degrees from a technological point of view? To answer this question, we suggest following the technological platform view.

### 3.1 Theory

The scientific literature uses the platform concept in at least two distinct ways, i.e., as a form of market organization (e.g., Refs. [[45], [46], [47]]) or as an engineering design approach (e.g., Refs. [[48], [49], [50], [51], [52]]).

The former interprets platforms as businesses exploring network effects [45,47] and reducing transaction costs [46] of human interaction. In this view, platforms are considered to be hubs for value exchange [53], organizing two-sided or multisided markets [54,55] that exert positive network effects (e.g., Refs. [56,57]).

The latter uses the platform term as a label for hierarchical modular product architectures [48,50,51]. The present study refers to the product architecture view of platforms solely, i.e., a purely technological view.

While there are different definitions of what constitutes a technological platform (e.g., Refs. [[49], [50], [51], [52],^58^,^59^]), all these definitions share the commonality of systematic reuse of components across different products within a product family [48].

For the purpose of our study, we define platform architecture by modularity in combination with hierarchical ordering of modules. On an abstract level, modularity can be interpreted as a very general set of principles for managing complexity; complex systems are broken up into discrete, manageable pieces that can then only communicate with each other through standardized interfaces [60]. On a more applied level, the construct of modularity has been comprehensively discussed from different perspectives. In fact, Salvador [61] identified 40 different definitions of modularity. Such definitions are based on one or more of the following five criteria: component commonality, component combinability, function binding, interface standardization, and loose coupling. We omit the discussion of the component commonality and loose coupling criteria as these are irrelevant for our following discussion.

According to component combinability, modularization means that different product configurations can be obtained by mixing and matching components taken from a given set. For example, in automobile construction, one may use five different motor types and combine those with three different car body types to obtain 15 different car designs. The component combinability view stresses the opportunity to maximize the combinational variety of assemblies from a given set of parts [61].

According to the function binding criterion, a module is defined as a component that carries out one or more specific functions on its own. Thus, modular products do not embed function sharing, i.e., each function is performed by just one module. For example, a power drill is usually just a motor, and its function is to drive whatever other function module is plugged in. Only if a drill bit is plugged in does the power drill really become a drill. The function of the drill bit itself is to drill, which cannot be performed by the motor module or other modules such as grinders or saws. Function binding is not necessarily limited to a 1:1 relation between functions and modules; one module could provide different functions. It is just that one function is not shared among different modules. If single modules provide more than just one function, function binding is labeled as packaged function binding [61].

The interface standardization criterion refers to the necessity to standardize the interfaces of different modules so that the modules can be combined and used together. If the drill bit does not fit into the power drill, then both modules are rather useless. The interfaces constrain the dependencies between modules [62]. There must be standards for how to exchange materials between modules (e.g., how to get fuel from the tank into a motor), how to exchange information (e.g., between processors and software), how to transfer energy (e.g., electricity from a power plant to your radio), or how modules spatially interact (e.g., by standardizing pipe diameters) [61]. Modularity, along with its standardized interfaces, enables the reduction of the costs of splitting design and production across multiple firms [62,63].

For our study on the nature of educational degrees, we define modularization as a concept based on the aforementioned three criteria. First, total education is divided into functionally distinct educational modules. We thus refer to the function binding criterion to define modularity. Educational modules are defined by the different types of degrees, from school to university degrees. Each degree is constructed as a module that ideally provides a package of functions, where different degrees provide different packages.

Second, we refer to the component combinability view. Accordingly, modularity implies that modules can be mixed and matched. In degree architectures, this is reflected, for example, in the opportunity to mix a high school degree with any degree in higher education or to mix a degree in engineering with one in law to become a patent lawyer.

Third, our working definition of modularization also includes the standardization of interfaces. According to that view, modules become modules only if their interfaces are standardized so that modules can be combined. Within a platform architecture, different modules communicate with each other only through standardized interfaces [60]. The interfaces establish the boundaries of modules. In the educational context, this means, for example, that the exchange of information in higher education is not hindered by the fact that the student cannot read. The functionality of being able to read is assigned to the school education module and can thus be expected to be present when a student with a high school diploma enters college. The higher-level degree neatly “plugs” in.

Modularization does not necessarily refer to the production side alone. If interfaces allow to do so, customers themselves might mix and match modules according to their own preferences [61], an aspect of modularization labeled by Baldwin and Clark [64] as “modularity in use”. Modularity in use is what we observe in educational choices where students mix their school education modules with higher-level degree programs according to their own preferences.

However, modularization is not sufficient to define a platform architecture. It further entails a hierarchical order of modules. Baldwin and Woodard [62] characterized platforms as a type of technological architecture that uses modularity not only as its main design feature but also as one that is structured around a core. In a platform architecture, there is thus an indispensable core module that must always be used together with any of the other peripheral modules [62]. The stable, low-variety core constitutes the platform [65], i.e., the core is the platform. It is also the core module that establishes the interfaces of the system governing the interactions among different modules. These interfaces must be stable in relation to the components so that interfaces must be part of the platform [62]. In software architectures, the operating system can be interpreted as the core, whereas the applications are the peripheral modules [66,67]. Herein, the operating system specifies the interfaces.

Thus, a modular design becomes a platform architecture only if it is hierarchically structured around a core. For example, a cutlery set is modular in that a fork has a distinct function from a knife or a spoon. However, because all can be used independently, cutlery sets have no indispensable core and thus do not represent a platform architecture.

The modules within a platform architecture differ in terms of variability. The core is a relatively stable module, whereas the peripheral, complementary modules are much more variable [62]. Complements, i.e., complementary modules, describe goods and services built on a platform [65,68].

The stable core components support variety and evolvability by constraining the linkages between the other components [62]. The value of the core good is higher for adopters if some complement is also obtained [69]. Indeed, an operating system, i.e., the core of a software architecture, has little to no value on its own until it is complemented by applications [[69], [70], [71]]. Reusing the core module across a product family allows for economies of scope in production [48]. For example, it has been reported that a switch to the product family approach has cut product costs by 50 % at Black and Decker and by 25 % in the manufacturing of agricultural and industrial equipment [72].

The technological platform design is not necessarily limited to a two-level approach; multilevel designs are also possible. For example, a software architecture has more than the two layers of an operating system and some applications. Below the operating system level, there is, of course, the hardware. On top follows the drivers, the operating system, the so-called middleware, and then the applications [73].

The platform concept has been applied to within-firm production systems, across-firm supply chains, and ecosystems [48]. A platform ecosystem describes the platform and its network of complementors that produce complements to enhance platform value [74]. Complementors are the providers of complementary products to mutual customers [70]. In education, colleges and universities complement the core module school education.

Overall, technological platforms are considered to be successful if network effects are purposefully exploited [48]. It is important to establish a community of consumers that is attracted to the provision of complementary products being offered by developers [65]. To ensure platform growth, the platform itself should also be characterized by a specific degree of openness so that developers can contribute new complements considering the constant functionality of the core [48]. In fact, educational ecosystems are quite open, as colleges, universities, and other producers are free to offer degrees according to their own and students’ preferences.

#### 3.1.1 Scope

According to Wheelwright and Clark [50], platforms are supposed to enable easy modification so that features or modules may be added, substituted, or removed. In this context, each module has a limited scope of functionalities which, in turn, characterizes that module [50].

However, which specific functions are included in the core or are assigned to peripheral complements can be decided by platform owners based on their individual interests [75] while also depending on the infrastructure surrounding the platform [76]. Thus, the assignment of functionalities is not generalizable across all types of platforms [76]. As a rule, reusable and stable components are assigned to the core, whereas variable components are assigned to the complements [62]. If a platform is about to be newly established, companies may individually design fundamental elements that are then shaping the core of an embryonic platform. This procedure is often referred to as coring. It integrates those components into the core that provide solutions to technological issues affecting a wide range of the remaining parts of the system [71]. For example, reading, writing, and basic math skills are assigned to the core school educations as these functionalities affect those of all peripheral educational modules.

Furthermore, an already existing core may be expanded by adding new functionalities. For example, millions of lines of code continuously add new functionalities to operating systems over time, such as the Linux kernel [77]. In education, adding new functionalities is, however, much more limited than in software architectures. Adding new lines of code to an operating system comes at marginal costs, as the tremendous improvements in hardware made such additions feasible. The same is obviously not true for students’ brainware.

#### 3.1.2 Quality, standardization, and compatibility

The platform architecture governs the interactions among the ecosystem's components [62]. In this context, technical standards are considered to be rules or norms describing a given product's performance, safety, or quality [78] and defining the platform's technological specification. In this way, standards ensure compatibility [79] and a consistent quality of the components of the ecosystem. From a user's point of view, standardization resulting in compatibility between different modules is particularly preferable if the user is interested in obtaining a portfolio of modules, if the single modules are rather expensive and long-lived, and if there are no low-cost converters or adaptors [80]. With respect to school education, these criteria are met. School education is a lifelong investment. If that investment does not meet compatibility standards, there are no low-cost converters turning one's incompetence into Ivy League access.

#### 3.1.3 Modular hierarchical embeddedness

The modules within a platform architecture are never stand-alone modules as they are always embedded. Embedding and using core components enable the realization of several striking advantages, such as economies of scale through increased production volume, amortization of fixed costs across product families or generations, and more efficient use of complementary assets such as distribution channels and technical support services [62]. The modular design allows for testing many product variants and selecting only the best ones without compromising the whole system [63]. The split between the stable core components and the variable peripheral components enables the realization of economies of scope at the system level. Economies of scope are created by a cost reduction of developing product variants targeted at different markets or incorporating new technologies [62].

#### 3.1.4 Summary

To summarize, a platform architecture is a modular product design consisting of a relatively stable core and a much more variable periphery of complementary modules. Each module, i.e., the core and each complementary module, is defined by the scope of functionalities offered by the respective module. The modules are embedded in a hierarchically ordered architecture structured around the core. One cannot use any complement without installing the core first. The core specifies and provides functionalities that can be used to build complements upon. To ensure compatibility among the components of the ecosystem, the technological specification of the platform is defined by compatibility standards prescribed by the core.

### 3.2 Degrees and educational ecosystems: an applied technological platform view

In the following, we will argue that the design of degrees and their hierarchical embeddedness within an educational ecosystem closely resemble the architecture of product platforms. Therefore, we interpret school education from primary to high school as the core and study programs from colleges and universities as the peripheral complements. The degree of some school X can be perceived as the technological platform, i.e., the core, that serves as the basis to which college Y's next-level degree, i.e., the peripheral component, can be attached. A school degree is thus a foundation on which other providers of educational services can build related, higher-level degrees.

As outlined above, modules are defined by their functionalities, and they may have one or more of them. According to scope conditions $C_{1}$ and $C_{2}$, school degrees cover a clearly delimited scope of subjects, trying to install respective functionalities such as the competencies to read, write, or calculate. The school education module, like other modules, limits the number of functionalities covered. According to $C_{2}$, there is always an upper limit of courses that can be taken. Meanwhile, $C_{1}$ guarantees that those functionalities are present at least at a sufficient level. These properties of degrees resemble the aspect of modularity in platform product architectures.

The next observable resemblance between degrees within their educational ecosystems and technological platforms is their hierarchical design. According to condition $C_{5}$, a higher-level degree can only be obtained after earning all lower-level degrees. This exactly reflects the core complement architecture of platform designs. There is, however, one caveat. Within educational ecosystems, this is just a rule-based restriction rather than a definite technological necessity like in product architectures. The hierarchical structure of technological platforms is driven by nonnegotiable technological necessity. An application cannot run without an operating system, and an operating system cannot run without drivers and hardware. This may be different in educational ecosystems. Eventually, an individual without a school degree may be able to meet the intellectual requirements of a college degree, and an individual without any educational degree may still be able to deliver a work that would be worth a PhD. However, chances for such attainments are small; thus, a rule requiring the acquisition of all lower-level degrees before entering a higher-degree program seems quite sensible. In that sense, $C_{5}$ can also be perceived as a condition that is imposed out of the technological necessity of a platform design.

As outlined above, the use of core components enables the realization of several advantages, including economies of scale through increased production volume, amortization of fixed costs across product families or generations, and more efficient use of complementary assets such as distribution channels or technical support services [62]. The production and distribution of school degrees exhibit such economies of scale on the provider side. The standardized composition of school degrees reduces the cost of developing degrees. The distribution of degrees through schools as awarding institutions further enables the use of the distribution channels for multiple generations of students. In addition, teachers’ training allows for long amortization times of training costs. Furthermore, the standardization of school degrees allows for the standardization of teacher education.

Platform designs critically focus on compatibility between the core and the complements. Thus, the standardization of interfaces is sine qua non. School degrees are in fact highly standardized. One approach of standardization is to define course sets. Within degrees, there are ex ante defined sets of mandatory and voluntary courses. School degrees mainly consist of mandatory sets. Restricting course sets is driven by the need to standardize functionalities of the core. If higher-level degrees need, for example, reading, writing, and some math functionalities, those functionalities must be provided by the core. From that point of view, it is conceivable that language and math courses belong to school education and are elements of the mandatory course set. Meanwhile, it is also not surprising that in higher-level degrees, the mandatory course sets are usually smaller than voluntary course sets.

However, human knowledge cannot be standardized (e.g., Ref. [81]) as accurately as technical components. This may lead to compatibility problems between the core and the complements. Here is where the applicability of the technological platform view to educational degrees seems somewhat limited. Students may leave their schools with a D-grade, but software companies would not sell operating systems of D-grade quality that are incompatible with their complements. While a difference exists, grading can simply be perceived as an institutional answer to this problem of nonstandardizability of human knowledge and capabilities. In that sense, grades are used like trade classes for agricultural products that also cannot be completely standardized. Grading helps to determine which complements, i.e., higher-level degrees, can and cannot be attached to the core.

The presumably biggest difference between product platforms and educational ones is the incentive problem inherent in human endeavors. A technical product never lacks motivation. An operating system has the functionalities given by its lines of code. It does not decide to cease assuming some functionality just because it feels exhausted or bored. These are human problems that must be accounted for only in an educational platform. One way of doing so is to use an incentive scheme, which could be a grading scheme, provided that better grades lead to better consequences. One of the better consequences may be access to better, more rewarding, higher-level degrees as described by $C_{5}$ and $C_{6}$.

Nevertheless, with respect to standardization, a major difference between technological platforms and a school degree platform remains. The generic ideal of a platform concept is that any peripheral module runs on the core. Regardless of whether your mobile works with iPhone Operating System or Android, the idea is that any application offered really runs on that system. In fact, applications conforming to the compatibility standards also run on your device in practice. The generic ideal of a platform architecture and what is achieved in practice coincide.

This is not the case in educational platforms. While school education is also meant to provide the basis for any higher-level educational endeavors, such a generic ideal is not achieved in practice. The standardizability problems make it impossible for the practical application to meet the generic ideal. It is thus questionable whether such an ideal of school education should be upheld. On the one hand, high-performing students attending an excellent high school achieve the ideal. They are prepared for whatever higher path they want to take. On the other hand, low-performing students leave schools too often without being prepared for even one higher-level option. This calls for a different treatment of higher-versus lower-performing students beyond giving them different grades. We suggest a basic design approach to address this problem in the following.

That fact that grading and certification of grades take place not only at the degree level but also at the course level, as depicted by $C_{4}$, addresses possible compatibility issues at the level of single functionalities. If a college application only requires math functionalities to be successfully installed, high school math grades reveal more important compatibility information than HSGPA. It thus makes sense to grade performance on an individual course level and to convey the results through transcripts of records.

To summarize, there are strong arguments suggesting that the design principles of degrees within their ecosystems follow the principles of technological platforms. If degrees are interpreted as modules within a technological platform design, then school education constitutes the core and is thus the most important module of the whole system. Inefficient cores compromise the overall performance of the educational ecosystem much more than any deficiencies of any complements ever could. In the next section, we use the platform view to address questions of optimizing the core design.

## 4 Optimizing the core

In the following, we use our platform view of degrees to analyze possible opportunities for school education improvements, i.e., the core of the educational ecosystem. As outlined above, modularization is at the heart of any platform architecture. Baldwin and Clark [64] provided a guide to modularity and referred to three design rules for an effective modular design.

(i) There needs to be an architecture that specifies what modules will be part of the system and what functions these modules shall have.
(ii) There need to be interfaces that describe how the modules will interact.
(iii) There need to be standards for testing a module's conformity to the design rules.

An implicit assumption behind rule (iii) is that if a module does not conform to the design rules and thus does not provide the assigned functionalities and/or meet the interface standards, it has to be redesigned until it meets the requirements.

Here, we will only discuss design rules (i) and (iii) with respect to the core module school education. Specifically, we will address the following two interrelated questions.

• What functionalities should belong to the core?
• Considering the incomplete standardizability of human knowledge, how can these core functionalities be implemented best?

With respect to the latter question, best implementation means that the functionalities can be counted on under all conceivable conditions, indicating, for example, that there are no relevant interface compatibility problems. The technological platform view now provides an inspiring way to address these questions.

We start by addressing the first question: What functionalities should belong to the core? As discussed above, on an abstract level, the core should integrate those components/functions that affect a wide range of the remaining parts of the system [71]. On an applied level, the core should provide such functionalities maximizing the value of the core for students. School education should provide those fundamental functionalities needed to perform well in the next-level programs of study and the subsequent periphery called life. These are the criteria to decide what belongs to the core and what does not.

We previously reviewed some of the overwhelming evidence that schooling works and that more education leads to better economic and life outcomes. However, such findings do not prove that school degrees are optimally designed. To design an optimal core, one needs to know the marginal economic and life quality returns of math, geography, and other potential candidates of the needed functionalities. Furthermore, one needs to know the synergies between literacy, numeracy, and other competencies as well as the marginal rates of substitution between courses such as history and geography for the given levels of life outcomes.

While it is striking how little evidence there is with respect to the consequences of letting children learn, for example, geography or history, at least some evidence pointing in the right direction is available. Among all functionalities that schools aim to provide, at least literacy and numeracy have been scrutinized somewhat in empirical work. For example, Chiswick et al. [82] found that about half of the effect of schooling on labor participation and unemployment can be attributed to those two competencies. Accordingly, it is demonstrated that above-average competencies lead to higher educational achievement, which, in turn, may result in leadership positions in various disciplines (e.g., Refs. [83,84]). In particular, literacy is indicated as a relevant prerequisite for social and economic successes, promoting an active involvement in society (e.g., Refs. [85,86]). In this regard, a longitudinal study conducted by Spengler et al. [87] reported that childhood literacy is positively correlated with educational attainment, income, and occupational status, even after 50 years. Other studies emphasized this relevance demonstrating that deficient reading skills pose an existential threat to further educational achievement and are associated with a significantly increased risk of unemployment and lower income (e.g., Refs. [[88], [89], [90], [91]]).

Furthermore, empirical work shows that numeracy has a crucial impact on educational achievement (e.g., Refs. [92,93]). In this context, several studies clearly state the high relevance of numeracy for career paths, which contributes to a positive effect on employment and a high impact on wages (e.g., Refs. [[94], [95], [96]]).

Despite numerous empirical studies reporting the significant importance of numeracy for educational and life successes, some findings suggest that certain mathematical competencies exhibit a decreasing relevance for educational and life successes over time. For example, Gaertner et al. [97] concluded that the completion of Algebra II in high school is more important for college than for career outcomes. They also found a decreasing relevance for students over time. In addition, Kim et al. [98] found that the completion of Algebra II increased the likelihood of attending college but did not have a significant impact on degree attainment. Furthermore, by analyzing long-term educational outcomes, Aucejo and James [99] found that the impact of verbal skills is at least double that of numeracy skills on university enrollment. While they found no effect of numeracy skills on verbal skills development, a positive impact of verbal skills on numeracy skills was observed [99]. This indicates that language skills may be a much more important factor for educational and life successes than numeracy skills.

Beyond the empirical findings on literacy and numeracy, evidence concerning the relevance of other school subjects, such as chemistry, history, and geography, to subsequent educational and life successes are completely missing. Considering the tremendous amounts of money invested in school education worldwide and students’ time invested in these subjects, it is striking that there is such a complete lack of evidence on the effects of these investments. There is no robust evidence on the absolute or relative importance of other subjects/functionalities taught in school aside from literacy and numeracy. We conclude that because of the lack of empirical evidence, the first question of what belongs to the core and what does not cannot be answered at the moment.

As discussed above, deficiency in reading skills poses an existential threat to further educational achievement and is associated with a substantially increased risk of unemployment and lower income (e.g., Refs. [[88], [89], [90], [91]]). This finding alone suggests that there may be something fundamentally wrong with school education. One can obviously identify deficiency in reading skills as a fundamental life threat only if there is a sufficient number of students leaving school with such deficiencies. A core that cannot ensure that the most fundamental functionalities are properly installed needs to be redesigned according to the aforementioned design rules [64]. Thus, the question that needs to be answered is whether literacy problems can be solved. Evidence suggests that this is typically the case (e.g., Refs. [[100], [101], [102], [103]]). The same holds true for mathematical impairments (e.g., Refs. [[104], [105], [106]]). The overall picture is that poor language proficiency and mathematical difficulties need not be permanent. The task of the core is to address such problems.

We now proceed to the second question: How can core functionalities be implemented best? As knowledge cannot be standardized as well as technical objects, some compromises may be necessary. At this point, we need to change perspectives again. So far, we have mainly discussed degrees as a general, abstract concept. The general interpretation of a high school diploma is that it provides a platform that is ideally compatible with all existing higher-level degrees. However, in practice, it will not be possible for all students to successfully complete each of the existing higher-level degrees. Students with learning difficulties are highly unlikely to join any of the higher-level programs. However, from an individual's perspective, the ability to earn any higher-level degree is less important anyway. Instead, it is important for each student to be able to achieve at least one satisfactory higher-level degree upon schooling. Rather than being guided by a generic ideal that every student should have access to all available higher-level degrees, one could argue instead that it would be more important to guarantee that every student has access to at least one satisfactory higher-level degree. In the following paragraphs, we discuss the adjustments in the architecture of schooling that are needed to achieve transition from a generic concept of educational attainment to one that is viable for each individual student. How can it be guaranteed that each student has at least one viable higher-level option upon completion of high school?

In the remainder of our paper, we suggest a design approach that may help underachievers without compromising the quality of schooling for better students.

**Fig. 1:**
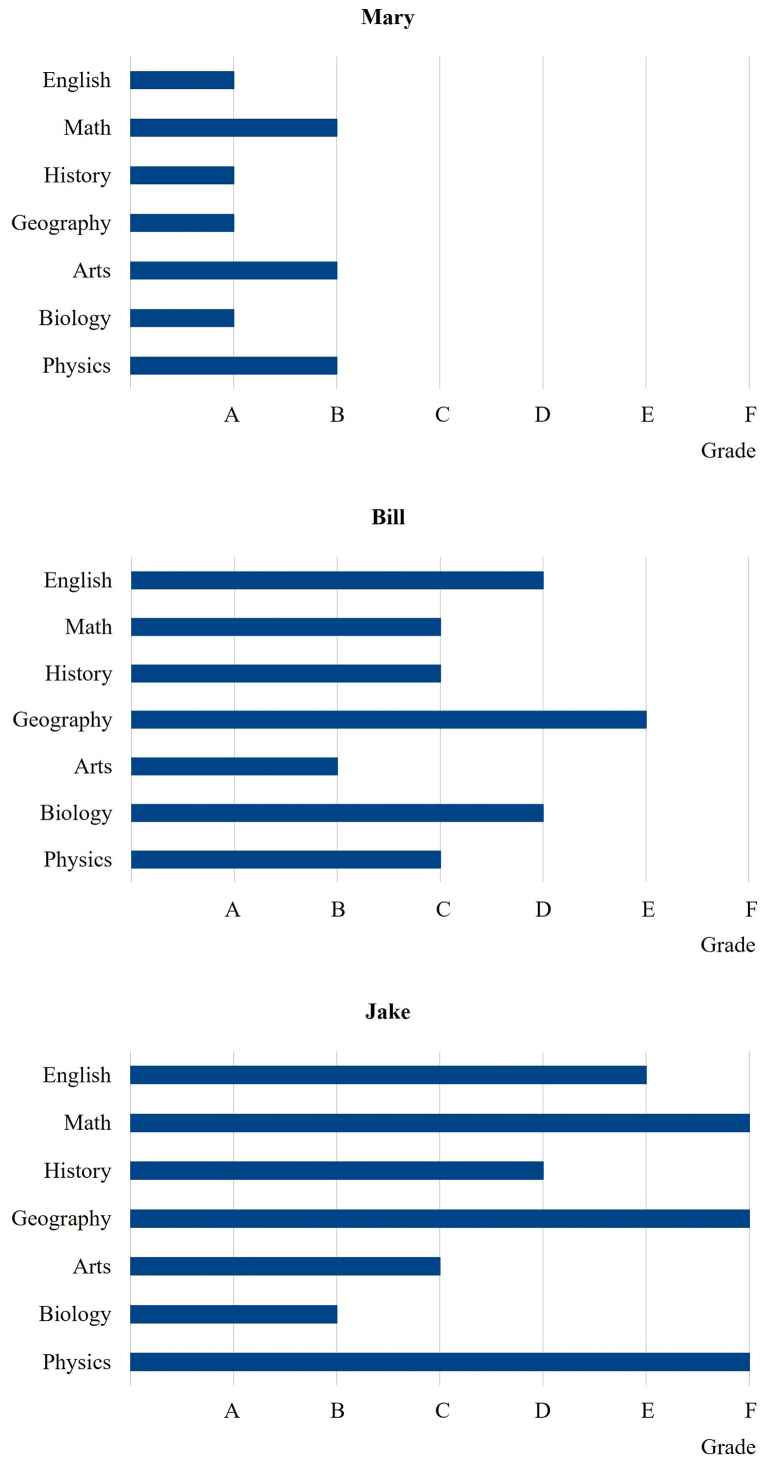
Conventional school degree design. Note: Fig. 1 shows the educational outcomes for three prototype students along the scope and quality dimensions according to the current school degree architecture.

$C_{1}$ to $C_{4}$ specify that degrees have basically two dimensions, namely, the set of different subjects covered and the quality of mastership of those subjects as measured by grades. In other words, degrees are characterized by scope and quality. Both dimensions could eventually be standardized to a greater or lesser extent. Fig. 1 presents the educational outcomes for three prototype students along the scope and quality dimensions according to the current school degree architecture.

In terms of standardization, conventional school education worldwide focuses solely on scope standardization. Similarly, quality problems, i.e., student performance deficiencies within subjects, are mainly accepted. Such differences are just accounted for through grading. Our prototype students Mary, Bill, and Jake have all studied the same subjects, but with highly differentiated quality outcomes. While Mary performs well, Bill's and Jake's examination results are worse and much worse, respectively.

**Fig. 2:**
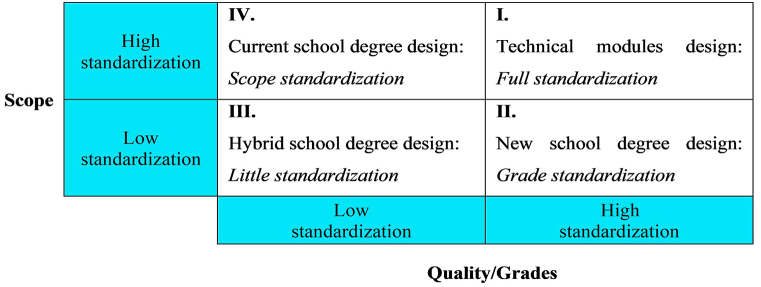
Feasible range of possible standardization approaches. Note: Fig. 2 shows possible standardization approaches with respect to scope and quality/grades.

Is scope standardization really the best possible approach? Fig. 2 presents the feasible range of possible standardization approaches. The modules of technical systems typically fall under full standardization quadrant I, i.e., scope and quality are fully standardized. For example, an operating system has a standardized scope of functionalities, and each functionality has a standardized quality.

However, humans and therefore human education are not standardizable to that extent; thus, quadrant I is reserved for technical modules only. Therefore, the design of educational degrees can only fall under quadrants II to IV. The current degree design of scope standardization is provided by quadrant IV, where scope is fully standardized and quality varies across students and subjects from exceptionally high to zero quality. It seems highly implausible for scope to be standardized instead of quality. As an analogy, scope standardization means that students are expected to learn many different musical instruments simultaneously. Furthermore, scope standardization is upheld even for students that are obviously not able to learn even a single instrument. Nevertheless, this design is dominating worldwide. Considering its dominance, this design is interestingly backed by no empirical evidence suggesting that it is a good or at least satisfactory design. In addition, it has never been tested against any design alternatives. Thus, the tremendous public and private spending on school education carries an enormous investment risk.

While we also cannot solve the empirical issue at this moment, we can at least provide an alternative design that could be tested against the dominating scope standardization. We suggest standardizing quality as measured by grades as far as possible and rather compromise on scope. If one buys an operating system, one would prefer the operating system with limited but well running functionalities over an operating system with a broad scope of error-prone functionalities.

We thus suggest switching from quadrant IV to II. Under a regime of quality standardization, one would start from a basic, minimum combination of subjects and standardize their quality as measured by grades. If students meet the grade standards, their range of subjects could be expanded. If their grades eventually deteriorated on the way to the final exams, the combination of subjects would be reduced again, eventually back to the minimum. The hybrid case of quadrant III differs from the suggested grade standardization only in that it would allow for a broader range of grades. This case is not discussed any further as it is only gradually different from the proposed grade standardization approach.

The conventional strategy of scope standardization leads to a situation wherein at the end of high school, some students have collected bad grades in a multitude of disciplines for a decade. They have learned nothing. Meanwhile, our approach suggests ensuring that at the end of high school, all students have about the same skills in basic subjects but eventually highly different skills in all the other subjects. This would mean that for those having trouble mastering the basic subjects (e.g., language and math), there are no additional subjects. Instead of geography and history, they receive additional training to improve their language and math skills. If math and language are the crucial drivers of academic and life successes, then the opportunity costs of geography for students having trouble with the former are simply too high. The task of the core within a technological platform is to provide functionalities that enable higher-level applications. If geography is not needed for that, it does not belong to the core of a troubled student. At best, it may serve as a complementary application for better-performing students.

We compare this grade standardization approach to the conventional one for our three prototype students. We assume that the design of this approach uses a grade standard for the basic subjects of B or better.

**Fig. 3:**
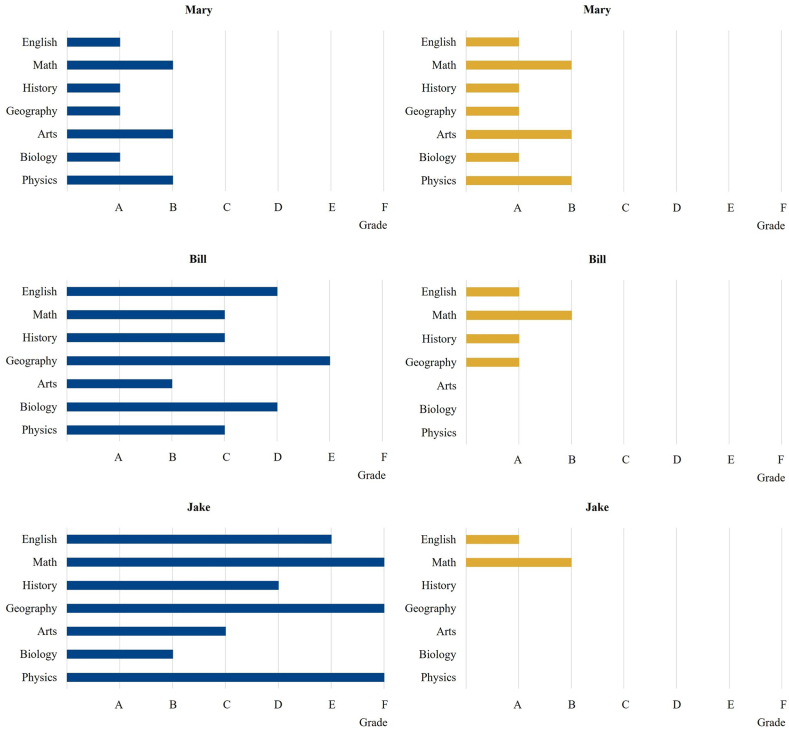
Comparison between scope and grade standardization. Note: Fig. 3 shows a comparison between the conventional scope standardization on the left side and the suggested grade standardization on the right side for the three prototype students.

As shown in Fig. 3, the new approach would not change anything for Mary. As she encounters no difficulties in any subject, she continues to attend the full range of subjects under the new approach. Bill, who has some trouble under the traditional scope standardization, would drop arts, biology, and physics and instead focus on English, math, history, and geography. Such a reduction in scope would be sufficient for him to meet the prescribed B standard in all the remaining subjects. The most profound change would occur with Jake, who struggles in almost all subjects under the old regime and thus limits his course selection to the basic English/math combination. This focus enables him to achieve at least a B grade in the basic combination. Because the functionalities of this basic combination are the most important and Jake greatly struggles in these subjects under the traditional scope standardization, Jake is likely to benefit the most from switching to grade standardization. He would receive more English and math instruction under the new system, which would enable him to keep up with his classmates in at least the basic combination. But he would, of course, fall short in all other subjects. However, because these subjects are less important and Jake would not have done well in them anyway, there is no real downside to missing out.

## 5 Discussion

We suggest exchanging the current scope standardization for the alternative grade standardization design of degrees.

This approach requires selecting an indispensable basic curriculum. As an example, we have suggested mathematics and the respective country's language as this basic curriculum. Beyond that, there is further empirical evidence of competencies that significantly improve students' academic performance and quality of life. These competencies make themselves worthy candidates for inclusion in the school core. For example, improved interpersonal communication has been consistently found to be associated with improved outcomes in almost all areas of life (e.g., Refs. [[107], [108], [109], [110]]). Thus, interpersonal communication as a school subject has much more evidence on its side than any traditional subject other than literacy and numeracy. Furthermore, there is some indirect empirical evidence that highlights the potential superiority of our suggestion. First, empirical research confirms the positive impact of instruction time on student performance (e.g., Refs. [111,112]). Cortes et al. [113] investigated the impact of an increase in instruction time in algebra on the associated course performance of ninth graders in Chicago public schools. Specifically, instruction time was doubled as part of an intervention aimed to help below-average performers in algebra. To keep students' total time requirements constant, additional algebra courses could be selected instead of other elective ones [113]. Doubling instruction time in algebra was then associated with improvements in corresponding test scores. In particular, students with comparatively lower proficiency in reading and algebra benefited most from the increased instruction time [113]. Taylor [114] found comparable effects when studying the impact of a double-dose algebra strategy implemented in Miami-Dade public schools for sixth graders. This strategy significantly improved the academic performance of the students, particularly the rather low-performing ones [114]. In these studies, the grades to be achieved have not been standardized as it would be the case in our proposed design. Nevertheless, a positive impact of increasing instruction time in a truly relevant subject was established. We expect that such an impact would be even stronger under our proposed grade standardization.

Implementing a grade standardization approach would not require higher time investments on the student's side. This would provide a significant advantage compared with the typical approach of additional instruction time for students with learning difficulties. In larger schools with many parallel classes, even teachers' total teaching hours would eventually be unaffected. The teaching hour demand for certain subjects, e.g., geography, would decrease, whereas that for language would increase. Ideally, the effects would offset each other.

When would our grade standardization approach outperform scope standardization? In general, our grade standardization scheme would be superior if two conditions were met.

Condition A indicates that more instruction within a single subject improves a student's performance in that subject. That, on average, is highly likely according to the evidence discussed. Condition B indicates that it is better to know more about less than to know less about more. Our expectation is that leaving high school with sound language and math competencies is better than leaving it with a broad scope of half-truths and pseudo-competencies. There is at least some evidence suggesting that condition B is also met. For example, Schwartz et al. [115] demonstrated that covering at least one relevant topic in high school science classes more extensively over a prolonged time period may lead to higher grades in college science courses. Similarly, Sadler and Tai [116] found evidence indicating that time-intensive coverage of less topics in high school physics courses predicts higher performance in college physics than covering a greater amount of content. These studies highlight the advantages of an intensive treatment of topics and the associated deepening of knowledge compared with a rather superficially acquired breadth of knowledge. Condition B is therefore also likely to be fulfilled. Considering the current state of knowledge, we therefore conclude that grade standardization is likely to be a highly superior architecture of schooling compared with the globally adapted scope standardization.

## 6 Limitations

Our suggested approach of grade standardization still has some limitations. First, our suggestion is purely theoretical. As it is not backed by empirical evidence so far, it remains somehow speculative. Grade standardization has not yet been adopted for schooling; thus, at this point, we are not able to prove the superiority of our suggestion empirically. Testing grade standardization would require a multiyear experiment. However, our suggested grade standardization is not more speculative than the current design of scope standardization. This holds true as scope standardization itself has never been put to a serious test. We still feel puzzled by the fact that scope standardization is applied worldwide without being supported by any single piece of evidence of its efficiency.

Furthermore, little is known about the relevance of subjects beyond literacy and numeracy; thus, the optimal basic curriculum remains to be determined. Therefore, a further limitation of our analysis is the nonexistence of empirical evidence identifying the truly relevant subjects. We think that it should be a truly worthwhile endeavor to identify which subjects should belong to the core and which subjects should be included in the basic combination of that core. It is possible that subjects like geography or arts should be transferred to the periphery.

Meanwhile, a focus on rather intellectual capabilities may not be warranted in all situations. For example, in some poorer countries, a relevant proportion of the rural population still operates subsistence agriculture. Students in such circumstances may profit more from improved competencies in agriculture.

Another limitation of our suggestion refers to the organizational problems of grade standardization. It requires giving up the usual class organization of schools. If students have different curricula, they obviously do not learn the same simultaneously. This may lead to smaller or larger problems of reorganization. Solving these problems will tend to be easier for large schools. Large schools with high numbers of students and teachers can organize different curricula for different students much easier than small ones. Technical facilities will also play a pivotal role in enabling grade standardization. If digital learning equipment is available, it would be much easier to let students learn different things simultaneously under supervision of the same teacher. In particular, badly equipped schools in poorer countries and smaller schools in remote locations may not be able to fully use our proposed grade standardization.

Our proposed grade standardization aims to improve the learning situation of weaker students without triggering adverse effects for higher-performing students. Essentially, higher-performing students would neither profit nor suffer from switching regimes. If the goal was to support higher-performing students, other programs would still be warranted.

The grade standardization approach aims to improve learning environments across a range of regular schools. Thus, it does not address special cases such as students with severe mental disabilities.

## 7 Conclusion

Degrees are presumably the most important institution in education markets worldwide. However, despite their high importance, they remain poorly understood within the economics of education. As our first contribution, we offer the first differentiated scientific definition of what constitutes a degree. A degree is defined by a quantity and quality dimension, i.e., the scope of subjects covered and grades achieved. Degrees are hierarchically embedded, indicating that higher-level degree programs can only be joined after at least one degree in each of all lower levels has been obtained.

We then surveyed the design concept of a product platform architecture. A platform architecture is a hierarchically ordered modular product design. Such a design consists of an indispensable core module and peripheral, complementary modules that can be attached to/plugged into the core. The interoperability of modules is ensured by standardized interfaces, where standards are defined by the core module. Modules themselves are characterized by the functions they provide. Considering their standardized interfaces, modules enable combinability, allowing for mix-and-match strategies to arrive at the final product.

As our second contribution, we formulated the first theory of educational degrees. We demonstrated that the design characteristics of degrees closely resemble those of a platform product architecture. Single degrees can be interpreted as modules within a platform architecture, where the school degree constitutes the core. School degrees can be combined with higher-level degrees and define the compatibility standards. A good high school education provides skills, e.g., reading, writing, and math. Higher-level degree programs can then be built on top of these skills.

The success of a platform architecture significantly depends on the quality of the core. An inferior core allows only inferior complements. Therefore, optimizing the core is of great importance in constructing a successful platform architecture. According to the current school education approach, a rather large number of different subjects are prescribed worldwide. Differences in student performance are accepted and translated into grade differentiation. Thus, it is the scope that is standardized. Contrary to scope standardization, we propose that competencies, measured then by grades, be standardized in the truly relevant subjects. Differentiation across students should be limited to the scope of the subjects taken. Ultimately, this approach should ensure that all students leave school with only good grades/competencies, but eventually a highly differentiated number of them. Our third contribution is the application of our platform theory of degrees to derive this fundamentally different school degree design.

Because the platform view highlights the importance of product architectures, new questions can be asked within this perspective, for example, does the previous traditional three-tier division into primary, secondary, and tertiary education still make sense considering the exponential growth of humanity's (scientific) knowledge (e.g., Refs. [[117], [118], [119]])? Would a four- or five-tiered architecture be more appropriate today? Should we accept or even enforce competition so that completely different cores are developed and tested? Henderson and Clark [120] argued that changes in product architectures can provide significant competitive advantages beyond those that can be achieved by improvements within an existing architecture. This may be especially true for the architecture of education as this architecture affects almost any human being.

## Ethics statement

Informed consent was not required for this study because this study is based exclusively on published literature.

## Data availability statement

No data were used for the research described in this study.

## Funding statement

This research did not receive any specific funding.

## CRediT authorship contribution statement

**Stefan Winter:** Writing – review & editing, Writing – original draft, Conceptualization. **Melissa Kistner:** Writing – review & editing, Writing – original draft, Conceptualization. **Deborah Maffia:** Writing – review & editing, Writing – original draft, Conceptualization.

## Declaration of competing interest

The authors declare that they have no known competing financial interests or personal relationships that could have appeared to influence the work reported in this manuscript.
